# Histological analysis of collagen composition in the stifle joint capsule of dogs with congenital patellar luxation and cranial cruciate ligament rupture

**DOI:** 10.1016/j.vas.2026.100635

**Published:** 2026-03-26

**Authors:** Mario Candela Andrade, Petra Peer, Pavel Slunsky, Matias Aguilera-Rojas, Johanna Plendl, Leo Brunnberg

**Affiliations:** a Department of Medicine, Health and Medical University, Potsdam, Germany; b South Tyrolean Health Authority, Veterinary Service, Health District of Brixen, Brixen, South Tyrol, Italy; c Deparment of Veterinary Clinical Sciences, Jockey Club College of Veterinary Medicine and Life Sciences, City University of Hong Kong, Hong Kong SAR, China; d Institute of Veterinary Anatomy, Center for Biomedical Sciences, School of Veterinary Medicine, Freie Universität Berlin, Germany; e Small Animal Clinic, Department of Veterinary Medicine, Freie Universität Berlin, Berlin, Germany

**Keywords:** Knee, Dislocation, Tissue, Dog, Immunohistochemical analyses, PL, CCLR, Stifle joint capsule

## Abstract

This study aimed to characterize collagen alterations in the stifle joint capsule of dogs diagnosed with patellar luxation or cranial cruciate ligament rupture. Tissue samples were obtained from affected dogs undergoing surgical correction, while control samples were collected from dogs with distal femoral fractures and unrelated conditions. Histological and immunohistochemical analyses were performed to quantify total collagen content and evaluate specific collagen types across the stratum fibrosum, stratum subsynoviale, and stratum synoviale. Collagen distribution varied significantly among capsule layers, with the stratum subsynoviale exhibiting the highest proportional collagen area, particularly in both proximal and distal regions. Larger breed dogs displayed less dense collagen networks compared to smaller breeds. Chronic joint disease was associated with elevated collagen types V and VI in the stratum synoviale, whereas control samples showed higher collagen type IV in the stratum fibrosum. Additionally, dogs presenting with peracute lameness exhibited markedly lower collagen type VI levels than those with subchronic lameness. These findings demonstrate distinct collagen abnormalities associated with patellar luxation and support the role of structural connective tissue defects in joint instability, strengthening the case for early detection and targeted therapeutic strategies.

## 1 Introduction

Congenital patellar luxation (PL) and cranial cruciate ligament rupture (CCLR) are among the most common hereditary musculoskeletal disorders in dogs (Wangdee et al., 2014; Farrell et al., 2015; Candela Andrade et al., 2020; Kowaleski et al., 2018). PL, typically medial, arises from a combination of skeletal malformations and soft tissue abnormalities such as joint capsule laxity. Likewise, CCLR develops through interacting factors including excessive tibial plateau angle, increased body weight, age, breed predisposition, and progressive ligament degeneration (Farrell et al., 2015; Candela Andrade et al., 2020; Kowaleski et al., 2018). Despite their prevalence, most PL studies have focused on clinical or radiographic features, with limited histopathological or molecular evaluation (Wangdee et al., 2014; Candela Andrade et al., 2020). In our previous work (Candela Andrade et al., 2025), we identified microstructural alterations in the stifle joint capsule of dogs with PL or CCLR, including changes in capsule thickness, villous formation, and loss of the stratum subsynoviale in chronic cases, as well as breed-related differences. However, the absence of immunohistochemical assessment of collagen architecture represented a major limitation, leaving unresolved questions regarding collagen organization, subtype distribution, and the molecular basis underlying the observed structural abnormalities. Collagen is a key structural component of connective tissues and plays a central role in maintaining tensile strength and joint capsule integrity. Alterations in collagen composition or organization can compromise joint stability, as demonstrated in joint hypermobility and chronic joint disorders in both humans and dogs (Paciello et al., 2003; Temwichitr et al., 2007; Hildebrand et al., 2005). Although collagen remodeling has been described in chronic joint disease, detailed characterization of collagen organization within the canine stifle joint capsule remains limited particularly with respect to its collagen composition and layer-specific organization in common orthopedic conditions such as PL and CCLR. In particular, layer-specific immunohistochemical mapping of collagen subtypes in dogs affected by patellar luxation or cranial cruciate ligament rupture has not previously been reported.

Building on our previous histomorphological findings (Candela Andrade et al., 2025), the present study aims to elucidate extracellular matrix remodeling in the canine stifle joint capsule associated with joint instability. To our knowledge, this study represents the first layer-specific immunohistochemical mapping of collagen subtypes in the stifle joint capsule of dogs with PL or CCLR. Using histological and immunohistochemical analyses, we evaluated collagen distribution and composition across distinct capsular layers in dogs diagnosed with patellar luxation (PL) or cranial cruciate ligament rupture (CCLR). The analysis primarily focused on collagen types I and III, which are critical for capsular tensile strength and tissue remodeling (Bey et al., 2005; Hildebrand et al., 2005), while additional collagen subtypes were assessed to provide a broader characterization of extracellular matrix alterations underlying capsular remodeling. Through this approach, we aim to provide new insight into extracellular matrix remodeling associated with joint instability.

## 2 Materials and methods

### 2.1 Patients

The patient selection, sampling strategy, and clinical criteria used in this study followed the protocol established in our previously published investigation of stifle joint capsule microstructure (Candela Andrade et al., 2025) (Supplementary Table 1) . In brief, the study population consisted of dogs undergoing surgical treatment for medially directed patellar luxation (PL) or cranial cruciate ligament rupture (CCLR), in which a small section of the joint capsule is routinely excised to perform lateral capsular imbrication. Dogs presenting with both PL and CCLR in the same limb were classified as a separate combined group. Control samples were obtained from dogs euthanized for unrelated medical conditions or from dogs surgically managed for distal femoral fractures in which an arthrotomy and capsular imbrication were required due to hemarthrosis. Written owner consent for the collection and use of joint capsule tissue was obtained in all cases.

Animals were assigned to the following groups:

I- Study groups:

PL group, comprising dogs diagnosed and surgically treated for PL;

CCLR group, consisting of dogs undergoing surgery for CCLR;

PL + CCLR group, including dogs with concomitant PL and CCLR;

II- Control group, consisting of dogs without orthopedic disease, confirmed through history, physical examination, and radiographic assessment, or dogs with distal femoral fractures requiring arthrotomy.

Diagnostic confirmation of PL was based on orthopedic examination and graded according to the Singleton system (Singleton, 1969). CCLR diagnosis relied on standard clinical indicators—such as joint pain, effusion, medial buttress formation, crepitus, and cranial drawer motion—supplemented by radiographic evaluation following the criteria outlined by Kowaleski et al. (2018).

Dogs of various breeds were included and classified according to the American Kennel Club breed standards (American Kennel Club, 1998). Weight categories were defined in 5-kg increments (0–5 kg up to >45 kg). Lameness duration was grouped as follows: peracute (1–2 days), acute (3–14 days), subacute (15–30 days), subchronic (31–90 days), and chronic (>90 days). Exclusion criteria consisted of incomplete medical records, inconsistent PL grading, inability to collect joint capsule tissue, or the presence of systemic diseases affecting joint integrity (e.g., polyarthritis, neoplasia, sepsis).

Control stifle joint capsule samples were obtained from dogs euthanized for severe, non-orthopedic medical conditions, with written owner consent for the use of tissues for research purposes. Two control animals were euthanized due to unrelated systemic diseases, including a severe intestinal rupture with a grave prognosis and end-stage chronic kidney disease. The absence of orthopedic disease was established based on clinical history, owner-reported information, and a comprehensive orthopedic examination performed prior to euthanasia. None of the control dogs showed clinical signs of lameness, stifle joint pathology, or had a history of orthopedic disease or surgical intervention. Control animals ranged in age from 0.3 to 15 years.

Due to ethical constraints and the limited availability of truly healthy stifle joint capsule tissue, age matching between control and affected groups was not feasible. Euthanasia was requested by the owners and performed according to welfare guidelines.

### 2.2 Examination material

A total of 59 stifle joint capsule samples were collected from dogs undergoing surgery for patellar luxation (PL), forming the "sick" group. Additionally, 11 samples were obtained from dogs with cranial cruciate ligament rupture (CCLR), 4 from dogs with both PL and concomitant CCLR, and 2 from dogs with distal femoral fractures. Two additional samples were obtained from dogs that had been euthanized for unrelated health reasons.

**Fig. 1:**
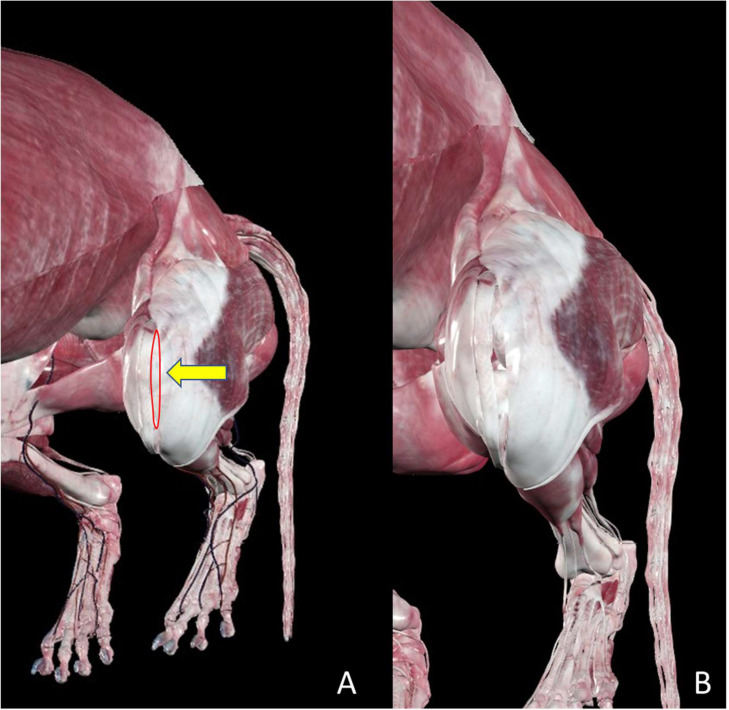
**A:** Illustration generated using a canine 3D model from the Anatomage Table (Vet Version 11). The red line indicates the lateral parapatellar approach and the location of the joint capsule section. **B:** Joint capsule appearance immediately after tissue resection before the imbrication.

The surgical sampling procedure followed the same protocol described in our previous publication (Candela Andrade et al., 2025) and in supplementary Table 2. Briefly, small portions of the stifle joint capsule were excised laterally and longitudinally using a scalpel (Aesculap®, Aesculap Inc., Center Valley, PA, USA) to obtain 0.5–2 cm² tissue sections. The incision was performed lateral parapatellar, with the proximal limit defined by the insertion of the vastus lateralis muscle into the joint capsule and the distal limit corresponding to the level of the distal patellar pole (Fig. 1). This approach allowed the execution of a lateral “capsular imbrication” as part of the therapeutic management of PL and CCLR cases, in which the capsule edges are overlapped or sutured to improve joint stability (Piermattei et al., 2006). Dogs treated for CCLR additionally underwent fascial imbrication following the Meutstege technique (Candela Andrade et al., 2020; Piermattei et al., 2006).

Samples were processed at the Institute for Veterinary Anatomy at Freie Universität Berlin for histological analysis. Following established guidelines (Mulisch & Welsch, 2010), the tissue samples underwent a series of preparation steps, including collection, fixation, dehydration, embedding, and sectioning to ensure optimal preservation (Supplementary Table 2). Total collagen content was assessed using the Volkmann–Strauss staining method under light microscopy, with multiple repetitions of each staining procedure to ensure reliability. A detailed description of this method is provided in Candela Andrade et al., 2025 and in supplementary Table 1. Histological sections were used for thickness measurements taken at 90 degrees to the inner surface of the joint capsule in the proximal, middle, and distal regions of the samples. This analysis involved defining the individual layers and their structural characteristics. Histomorphological criteria were applied to distinguish between the stratum synoviale, stratum subsynoviale, and stratum fibrosum within the respective sections of the joint capsule (Candela Andrade et al., 2025). Subsequently, immunohistochemistry was employed to differentiate the proportions of various collagen types across these layers.

### 2.3 Morphometry

Multiple histological and immunohistochemical sections were prepared from each of the 78 stifle joint capsule samples. Histological analysis included three slides per sample, each containing two stained sections to ensure reproducibility, while immunohistochemical procedures incorporated positive and negative controls for every antigen–antibody reaction. Microscopic evaluation was performed using a Zeiss Axioskop 40 microscope (Zeiss, Oberkochen, Germany) equipped with 1.25x, 5x, 10x, 20x, and 40x objectives. Image acquisition and quantification were carried out using NIS-Elements AR® software (Nikon, Chiyoda, Tokyo, Japan; Nikon, Düsseldorf, Germany). All images were captured under standardized illumination and exposure settings, which were kept constant throughout the study. The predefined color threshold for collagen detection was established prior to analysis and applied uniformly to all sections without case-specific adjustment. Regions of interest were selected systematically within predefined anatomical layers, avoiding areas of artifacts or sectioning defects.

**Fig. 2:**
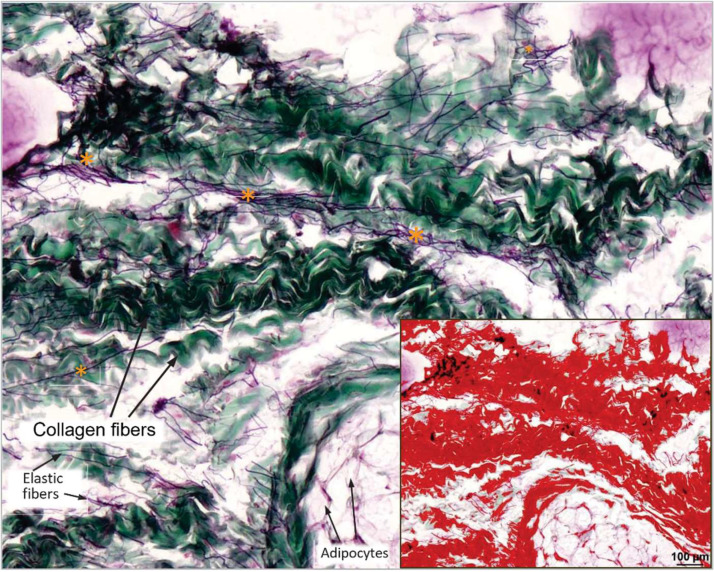
Collagen fibers (green) and elastic fibers (arrows and yellow asterisks) in the stratum fibrosum of a canine stifle joint capsule sample. The image analysis software NIS-Elements® marks the detected collagen fibers in red and subsequently calculates their surface area proportion (in %) relative to the entire image section. Staining method: Volkmann-Strauss; 10x magnification.

Collagen fiber surface-area proportions were determined using the Volkmann–Strauss method according to Mulisch and Welsch (2010), based on a predefined threshold for dark green–stained collagen fibers (Fig. 2). The software automatically quantified these values through color recognition. Measurements were obtained from six different 10x fields of view within each region, and the mean value was used for statistical analysis.

### 2.4 Immunohistochemistry

To compare connective tissue structure in healthy and diseased stifle joint capsules, immunohistochemistry (IHC) was used to analyze the distribution of collagen types I, III, IV, V, and VI. Table 1 summarizes the manufacturer information, antibody types, and optimized dilutions used for immunohistochemical staining of canine joint capsule samples. All antibodies are polyclonal and are suitable for use with formalin-fixed paraffin-embedded specimens.

**Table 1:** Antibodies used for immunohistochemistry (IHC) on canine joint capsule samples, along with corresponding optimized dilutions.

Antibody	Source/Catalog Nr.	Antigen Source	Used Dilution	Secondary Antibody, Concentration, Dilution
Polyclonal rabbit anti-Collagen I	Abcam/ ab34710	Rabbit serum	1/50	Donkey anti-Rabbit IgG (Abcam), 0.5 mg/ml, 1/300
Polyclonal rabbit anti-Collagen III	Abcam/ ab7778	Human and bovine placenta	1/100	Donkey anti-Rabbit IgG (Abcam), 0.5 mg/ml, 1/300
Polyclonal rabbit anti-Collagen IV	Abcam/ ab6586	Human and bovine placenta	1/20	EnVision+System-HRP labelled Polymer anti-Rabbit (Dako), ready to use
Polyclonal rabbit anti-Collagen V	Abcam/ ab7046	Human and bovine placenta	1/50	Donkey anti-Rabbit IgG (Abcam), 0.5 mg/ml, 1/300
Polyclonal rabbit anti-Collagen VI	Abcam/ ab6588	Human and bovine placenta	1/100	Donkey anti-Rabbit IgG (Abcam), 0.5 mg/ml, 1/300
Polyclonal rabbit anti-Collagen X	Abcam/ ab58632	Rat chondrosarcoma cells	1/100	EnVision+System-HRP labelled Polymer Anti-Rabbit (Dako), ready to use

### 2.5 Collagen type distribution in canine stifle joint capsules

To identify collagen types in the canine stifle joint capsule, tissue sections underwent deparaffinization using a graded alcohol series (maximum 3 min per step, except for two 10-minute xylene baths), followed by rehydration in buffer. After two 5-minute rinses in Tris-buffered saline (TBS) buffer, antigen retrieval was performed using the methods detailed in Table 2, optimized for each antibody.

**Table 2:** Steps and details for differentiation of collagen types in the canine stifle joint using immunohistochemistry, optimized for each antibody.

Step	Details
**Antigen Unmasking**	**Collagen Types I, III, V, VI**: 35 min at 96 °C in 0.01 M citrate buffer, pH 6.0 (Medax, Neumünster, Germany) **Collagen Types IV, V**: 35 min at 96 °C in Retrieval Solution, pH 6.0 (Dako, Hamburg, Germany) **Collagen Type X**: 3 min in Trypsin/EDTA at 37 °C
**Rinsing**	Twice in TBS at room temperature for 5 min each
**Peroxidase Blocking**	20 min in peroxidase block solution (3% hydrogen peroxide in TBS)
**Additional Rinsing**	5 min in TBS, followed by 0.01% Tween 20 in TBS (Dako, Hamburg, Germany)
**Blocking**	20 min in blocking solution (Incubation dab with 3% BSA and 3% normal serum) For rabbit antibodies: 1% milk added to blocking solution
**Primary Antibody Incubation**	**Collagen Types I, III, IV, V, VI**: Overnight at 4 °C **Collagen Type X**: 1 hour at 4 °C **Concentrations and Diluents**: - Collagen I: 1/50 in Incubation Buffer A - Collagen III: 1/100 in Incubation Buffer A - Collagen IV: 1/20 in Antibody Diluent (Dako, Hamburg, Germany) - Collagen V: 1/50 in Incubation Buffer A - Collagen VI: 1/100 in Incubation Buffer A - Collagen X: 1/100 in Incubation Buffer *A* + 0.5% milk
**Negative Controls**	**Collagen Types I, III, IV, V, VI**: - Rabbit-IgG, 1/2000 or 1/1000 (for Collagen VI) in Incubation Buffer A - Incubation Buffer A **Collagen X**: - Rabbit normal serum, 1/100 in Incubation Buffer *A* + 1% milk - Incubation Buffer *A* + 1% milk
**Secondary Antibody Incubation**	45 min at room temperature with Donkey anti-Rabbit IgG-HRP, diluted 1/300 in Incubation Buffer A For Collagen Types IV and X: EnVision+System-HRP labelled Polymer Anti-Rabbit (Dako, Hamburg, Germany)
**Visualization**	Diaminobenzidine (DAB) reaction solution for 10 min in the dark
**Counterstaining**	Mayer's hematoxylin (12)
**Double Staining**	First staining completed, LinBlock® used to remove the first antibody, followed by second detection with DAB for the first color reaction and Histogreen® for the second

### 2.6 Preliminary tests and controls

Antibody specificity was validated using both manufacturer-specified positive controls for the designated species and canine positive controls, taking advantage of cross-reactivity where applicable. The intensity and localization of staining in positive controls were assessed and compared with manufacturer specifications and relevant literature to confirm specific collagen labeling. Regular double-negative controls, which involved omitting both the primary and secondary antibodies, were conducted to identify any non-specific binding. This approach helped eliminate false-positive staining resulting from immunological cross-reactions or other tissue interactions with the immunohistochemical reagents. Optimal dilutions for collagen detection in dogs were determined and are detailed in supplementary Table 3. Comparative analyses were performed on canine tissue samples alongside the manufacturer-supplied positive controls to evaluate reaction strength. Despite numerous attempts, reliable detection of collagen type X was not achieved, even with various positive controls.

### 2.7 Evaluation of antigen presentation

**Fig. 3:**
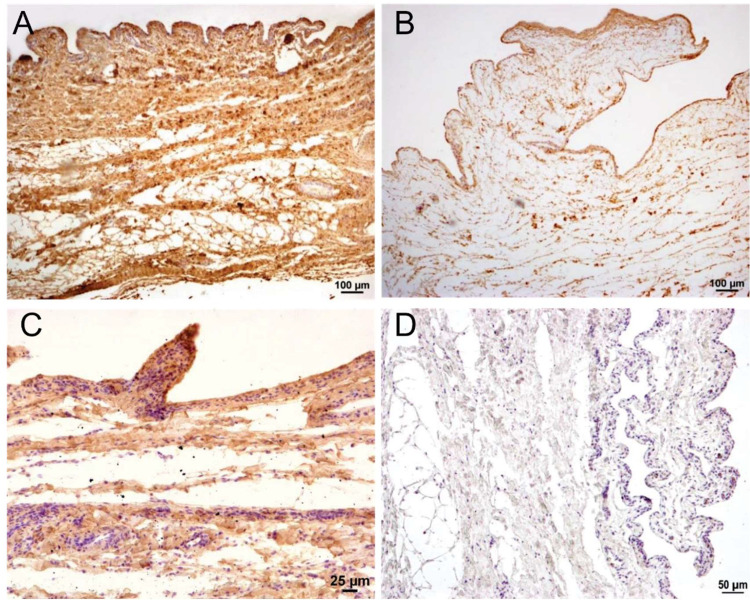
Semiquantitative scores for the presence of the examined collagen types in the canine stifle joint capsule. Fig. 3A shows a strongly positive marking (Score = 3), Fig. 3B shows a moderately positive marking (Score = 2), and Fig. 3C shows a weakly positive marking of Collagen Type I (Score = 1). Fig. 3D shows its negative marking (Score = 0). Counterstaining was done with Mayer's hematoxylin. Original magnification: A and *B* × 10; C and *D* × 20.

Immunohistochemical staining for collagen types I, III, IV, V, and VI was evaluated semiquantitatively using a four-point ordinal scale (0–3). A score of 0 indicated absent or non-specific staining, such as faint light brown reactions in muscle fiber bundles (Fig. 3D). Scores of 1, 2, and 3 corresponded to low, moderate, and high staining intensity, respectively, of collagen fibers (Fig. 3A–C). This semiquantitative analysis provided ordinal data assessing the relative abundance and spatial distribution of each collagen type within defined measurement areas.

Semiquantitative scoring was performed independently by two observers (including the second author). In cases of discrepant evaluations, the sections were jointly reviewed with a third investigator, and a consensus score was assigned. The findings are summarized in Table 3.

**Table 3:** Detected antigens, location, evaluation of detection.

Detected antigens	Assessment locations	Evaluation of detection
	proximal stratum fibrosum	
**Collagen Type I**	middle stratum fibrosum	Score = 0: negative
**Collagen Type III**	distal stratum fibrosum	Score = 1: positive
**Collagen Type IV**	proximal stratum subsynoviale	Score = 2: moderately positive
**Collagen Type V**	middle stratum subsynoviale	Score = 3: strongly positive
**Collagen Type VI**	distal stratum subsynoviale proximal stratum synoviale middle stratum synoviale distal stratum synoviale	

### 2.8 Statistical analysis

Data were analyzed using IBM SPSS Statistics® software (Version 22 for Windows; Armonk, NY, USA). The Kruskal-Wallis ANOVA with post-hoc testing, the Mann-Whitney test, and Bonferroni correction were employed to assess significant differences and correlations among the measurements across various animal groups. P-values of ≤ 0.001 were considered highly significant, those ≤ 0.01 as moderately significant, ≤ 0.05 as minimally significant, and values > 0.05 as not significant. Descriptive statistics were compiled in tables, and results were illustrated using boxplots, bar charts, and area charts.

## 3 Results

Epidemiological and clinical data regarding the patient sample, including age distribution, breeds, sex, weight classifications, type of disease, duration of lameness, grade of patellar luxation, distinction between unilateral and bilateral patellar luxation, degree of fibrin on the inner surface of the joint capsule, and the proportion of the area occupied by fibrin deposits, are detailed in the previous section of this research, explained in the results (Candela Andrade et al., 2025) and in supplementary Table 2.

### 3.1 Correlation between the presence of the stratum subsynoviale and increased collagen content

In our previous histomorphological study (Candela Andrade et al., 2025), the stratum subsynoviale was absent in the middle section of the stifle joint capsule in the majority of cases (59/78; 75.64%). Only 19 samples (24.35%) exhibited a clearly defined middle subsynovial layer. Comparative analysis using non-parametric Mann–Whitney U tests revealed significant differences between samples with and without a middle subsynovial layer in both the middle and distal sections of the stratum subsynoviale, as well as in the corresponding regions of the stratum synoviale. Notably, samples with a preserved middle subsynovial layer showed a higher mean collagen fiber area fraction (64.41%), which was positively correlated with collagen area fractions in the proximal and distal capsule regions (*P* = 0.001).

**Fig. 4:**
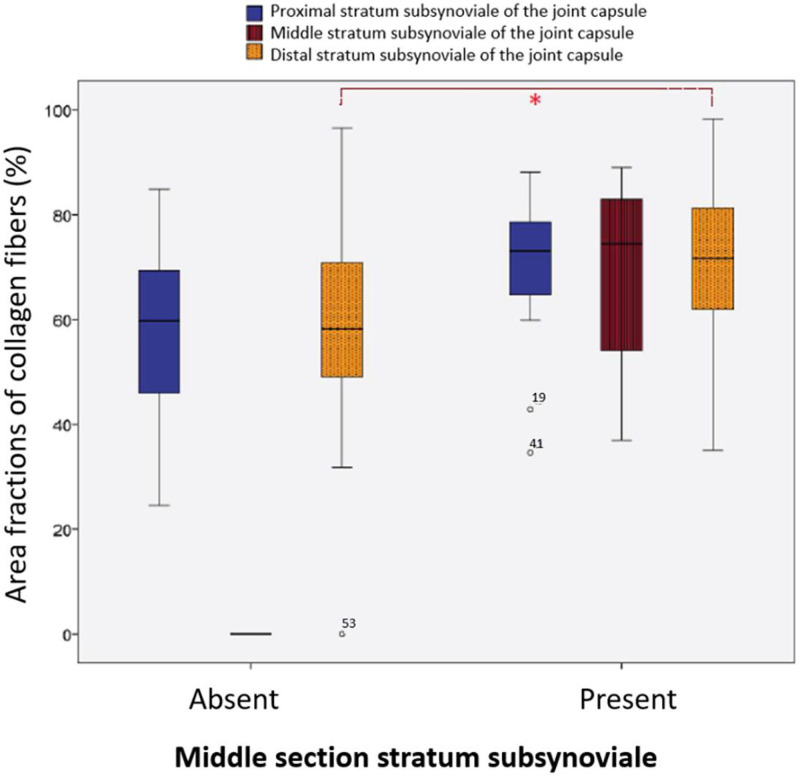
Area fractions of collagen fibers (%) in the three sections of the stratum subsynoviale in patients with and without a middle subsynovial layer.* Significance Level at *p* < 0.05.

In the distal portion of the stratum subsynoviale, significant differences were identified between the two groups (*P* = 0.020). In animals without a middle stratum subsynoviale, collagen fibers comprised an average of 58.11% of the total area, while in those with this layer, the fraction increased to 70.59% (Fig. 4).

**Fig. 5:**
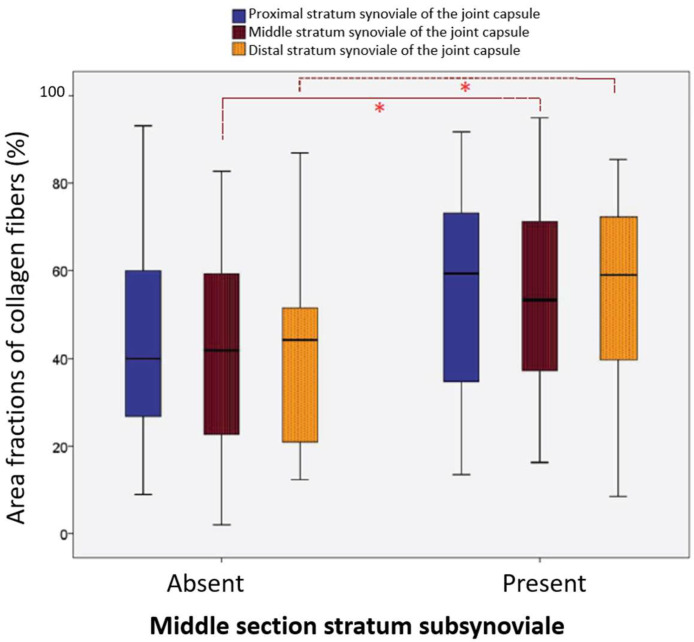
Area fractions of collagen fibers (%) in the three sections of the stratum synoviale in patients with and without a middle section of the stratum subsynoviale.* Significance level *p* < 0.05.

In the middle portion of the stratum synoviale (Fig. 5), dogs lacking a middle subsynovial layer displayed a collagen area fraction of 38.98%, which was significantly lower than the 53.48% found in dogs with this layer (*P* = 0.018). Additionally, in the distal region of the stratum synoviale, a significant difference of 12.85% was observed between the groups (*P* = 0.042, ANOVA). Dogs without the middle subsynovial layer had an average collagen area fraction of 41.03%, whereas those with this layer exhibited a higher fraction of 53.88% (Fig. 5).

These findings highlight significant variations in collagen content between dogs with and without a middle section of stratum subsynoviale across different regions of the joint capsule, indicating that the presence of this layer is associated with increased collagen content in both stratum synoviale and subsynoviale.

### 3.2 Area proportions (in %) of total collagen

Because the middle portion of the stratum subsynoviale was morphologically absent in 59 of 78 cases (75.64%), consistent with our previous observations in dogs with patellar luxation (Candela Andrade et al., 2025), this layer was excluded from further calculations of total collagen content (%), semiquantitative assessment of immunohistochemically detected collagen types within the joint capsule. Its inconsistent presence across specimens would have distorted area-based measurements and compromised inter-sample comparability, thereby introducing bias into quantitative analyses.

**Fig. 6:**
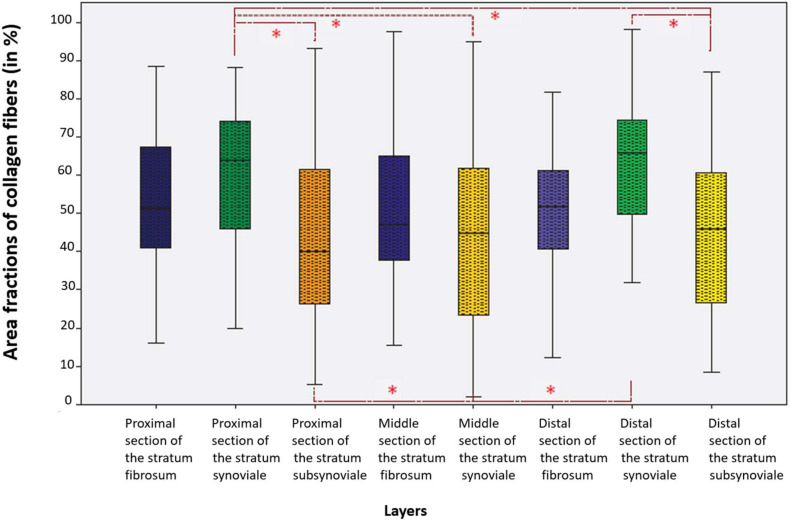
Area fractions of total collagen (%) across the different layers and sections of the stifle joint capsule.* Significance level *p* < 0.05.

Mean total collagen area fractions differed significantly among the nine measurement areas (Table 4). However, within each capsular layer, differences between proximal, middle, and distal sections were minimal, resulting in strong correlations across the three sections of the same layer (Fig. 6).

**Table 4:** Area proportions (%) of total collagen in the nine measurement areas. Abbreviations: SD-standard deviation.

Layer Section	Areal proportion of total collagen (in %)			
	Mean	SD	Minimum	Maximum
Proximal stratum fibrosum	53.62	17.05	16.20	88.50
Middle stratum fibrosum	51.22	18.66	15.50	97.60
Distal stratum fibrosum	51.19	16.72	12.30	81.75
Proximal stratum subsynoviale	61.11	17.27	19.90	88.15
Middle stratum subsynoviale	64.41	20.13	34.40	88.95
Distal stratum subsynoviale	62.03	18.45	0.00	98.30
Proximal stratum synoviale	44.04	22.34	5.20	93.10
Middle stratum synoviale	42.93	22.45	02.05	94.90
Distal stratum synoviale	44.92	21.90	8.50	86.90

In contrast, pronounced differences in total collagen content were observed between the three layers. The stratum subsynoviale exhibited the highest collagen area fractions (61.11–64.41%), followed by the stratum fibrosum (51.19–53.63%), whereas the stratum synoviale showed the lowest values (42.93–44.93%). Accordingly, significant positive correlations were identified between collagen area fractions in the stratum subsynoviale and the corresponding proximal, middle, and distal sections of the stratum fibrosum (*p* = 0.004 and *p* = 0.008 for proximal comparisons; *p* = 0.037 for middle–distal comparison; all Bonferroni corrected).

Pairwise comparisons further demonstrated that total collagen area fractions were significantly higher in the stratum subsynoviale than in the stratum synoviale within each corresponding proximal, middle, and distal section of the joint capsule (*P* < 0.001 for all comparisons, Bonferroni corrected; Fig. 6).

No significant associations were detected between total collagen area fraction and age, sex, body weight, disease type, lameness duration, patellar luxation grade, laterality (unilateral vs. bilateral), villous formation on the inner surface of the joint capsule, or the area proportion of fibrin deposits (all *P* > 0.05). However, medium-sized dogs (*n* = 19) exhibited a significantly lower total collagen area fraction in the proximal stratum fibrosum compared with large-sized dogs (*n* = 10; *P* < 0.05).

Taken together, total collagen quantity differed primarily between capsular layers but was not significantly influenced by disease state or lameness duration, indicating relative stability of overall collagen content across clinical conditions.

### Immunohistochemical analysis of collagen types I, III, IV, V, and VI

In contrast to the quantitative assessment of total collagen area fractions presented above, the following analyses focus on the semiquantitative immunohistochemical distribution of individual collagen types (I, III, IV, V, and VI). These evaluations address qualitative differences in collagen composition and localization rather than overall collagen quantity.

#### 3.3.1 Collagen type distribution across breed size groups

**Fig. 7:**
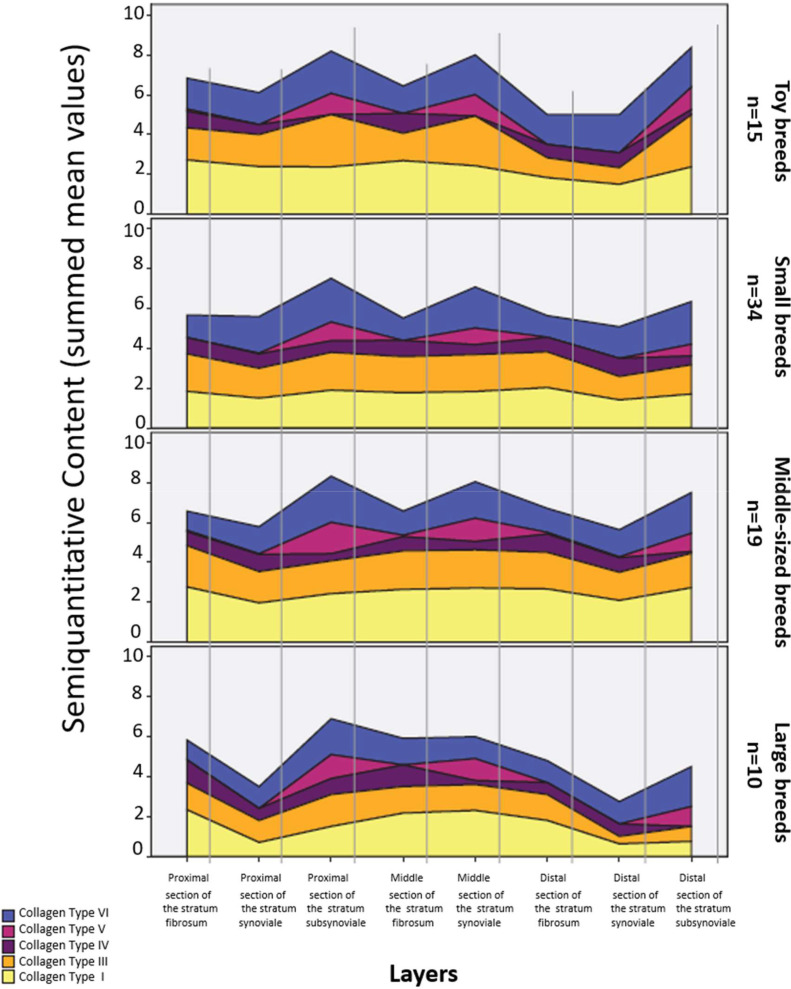
Semiquantitative content of the examined collagen types in the four breed groups.

The distribution of the examined collagen types varied among the four breed groups (Fig. 7). Non-parametric Mann–Whitney U tests for independent samples identified significant differences in nine measurement areas, which were further confirmed by one-way ANOVA.

Toy dogs demonstrated significantly higher collagen type I density in the proximal section of the stratum fibrosum (2.72) compared with small dogs (1.88; *p* = 0.050, Bonferroni). In the proximal stratum subsynoviale, collagen type I was significantly denser in toy dogs (2.39) than in large dogs (0.58; *p* = 0.011, Bonferroni). Additionally, collagen type III was significantly higher in toy dogs (1.87) than in large dogs (1.03; *p* = 0.047, Bonferroni). No significant differences were detected for collagen types IV, V, or VI in toy dogs relative to other groups.

Small dogs exhibited significantly lower collagen type I levels in the proximal stratum fibrosum (1.88) compared with toy (2.72) and medium-sized dogs (2.75; *p* = 0.007, Bonferroni), and in the middle section of the stratum fibrosum (1.87) compared with medium-sized dogs (2.66; *p* = 0.028, Bonferroni). No significant differences were observed for collagen types III, IV, V, or VI in this group.

Medium-sized dogs showed several differences in collagen distribution patterns. In the proximal stratum subsynoviale, collagen type I was significantly denser (2.00) than in large dogs (0.58; *p* = 0.011, Bonferroni). Similarly, collagen type III in the distal stratum synoviale was significantly higher (1.71) compared with large dogs (0.36; *p* = 0.016, Bonferroni). No significant differences were identified for collagen types IV, V, or VI.

Consistently, large dogs displayed significantly lower collagen type I content in the proximal stratum subsynoviale (0.58) compared with toy (2.39) and medium-sized dogs (2.00; *p* = 0.011, Bonferroni), and lower collagen type III levels in the distal stratum synoviale (0.38) compared with toy (2.19) and medium-sized dogs (1.71; *p* = 0.047, Bonferroni). No significant differences were observed for collagen types IV, V, or VI.

Breed-associated differences primarily involved collagen types I and III, whereas collagen types IV, V, and VI remained largely comparable across groups. In general, large dogs exhibited a less dense collagen fiber network than smaller breed groups, particularly for collagen types I and III. A comprehensive overview of these breed-associated differences in collagen distribution is provided in Supplementary Table 4.

#### 3.3.2 Collagen type distribution across different disease states

**Fig. 8:**
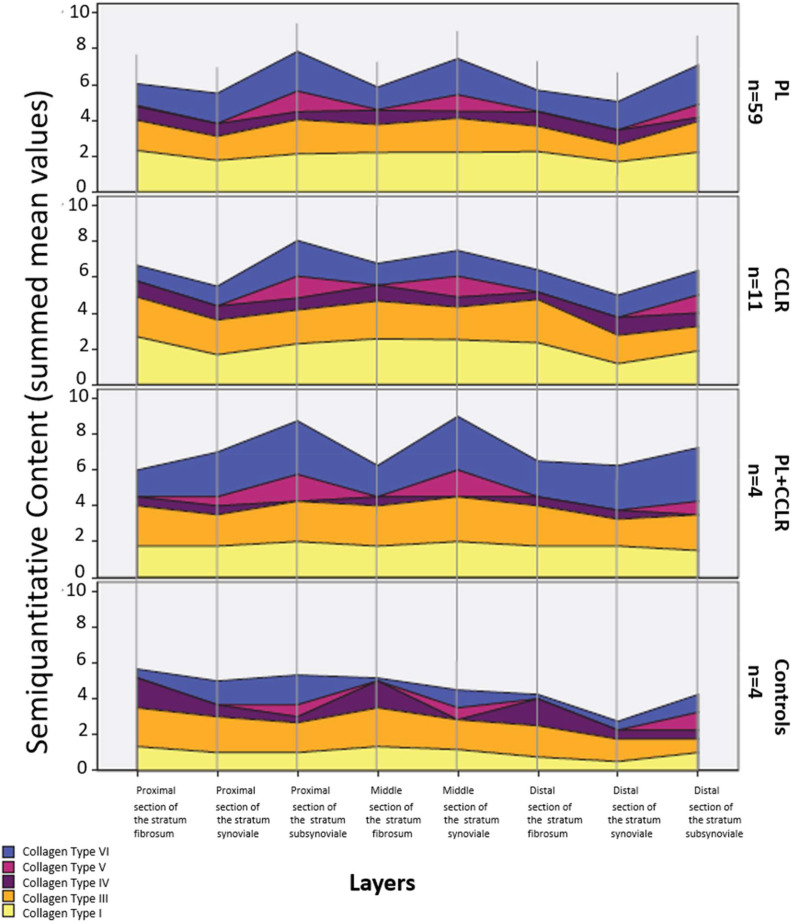
Semiquantitative content of the examined collagen types in the different diseases. Abbreviations: PL-patellar luxation; CCLR-cranial cruciate ligament rupture.

Collagen type distribution in dogs with patellar luxation (PL), cranial cruciate ligament rupture (CCLR), and the combined condition (PL+CCLR) showed broadly similar patterns that differed markedly from the control group (Fig. 8). In all diseased groups, collagen types I, III, and VI were consistently prevalent and distributed relatively uniformly across the layers of the stifle joint capsule.

Collagen types I and III were evenly distributed across anatomical sections in PL, CCLR, and PL+CCLR dogs. In contrast, control dogs exhibited significantly higher levels of collagen type III relative to type I across all layers. Collagen type IV was predominantly localized to vascular basement membranes and showed a generally uniform distribution across disease states. However, in the PL+CCLR group, collagen type IV was absent in the proximal and middle stratum synoviale and in the distal stratum subsynoviale, whereas controls demonstrated increased collagen type IV expression in the stratum fibrosum.

Collagen type V was predominantly concentrated in the stratum synoviale across all groups. Additional expression in the proximal stratum subsynoviale was observed exclusively in the PL+CCLR group. In this region, PL+CCLR dogs exhibited significantly higher collagen type V (0.33) compared with dogs affected by patellar luxation alone (0.01; *p* = 0.001, Bonferroni corrected). In contrast, control animals exhibited only minimal collagen type V expression in the proximal stratum subsynoviale, resulting in significantly lower levels compared with the PL+CCLR group (*p* = 0.002, Bonferroni corrected) (Supplementary Table 5).

Collagen type VI was most abundant in PL+CCLR dogs, followed by PL and CCLR groups, and lowest in controls. In the control group, a proximodistal decline in collagen type VI expression was observed (Fig. 8).

Overall, chronic joint pathology—particularly the combined occurrence of PL and CCLR—was characterized by increased collagen types V and VI within the stratum synoviale, whereas control dogs showed comparatively higher collagen type IV expression in the stratum fibrosum.

#### 3.3.3 Collagen type distribution and lameness duration

**Fig. 9:**
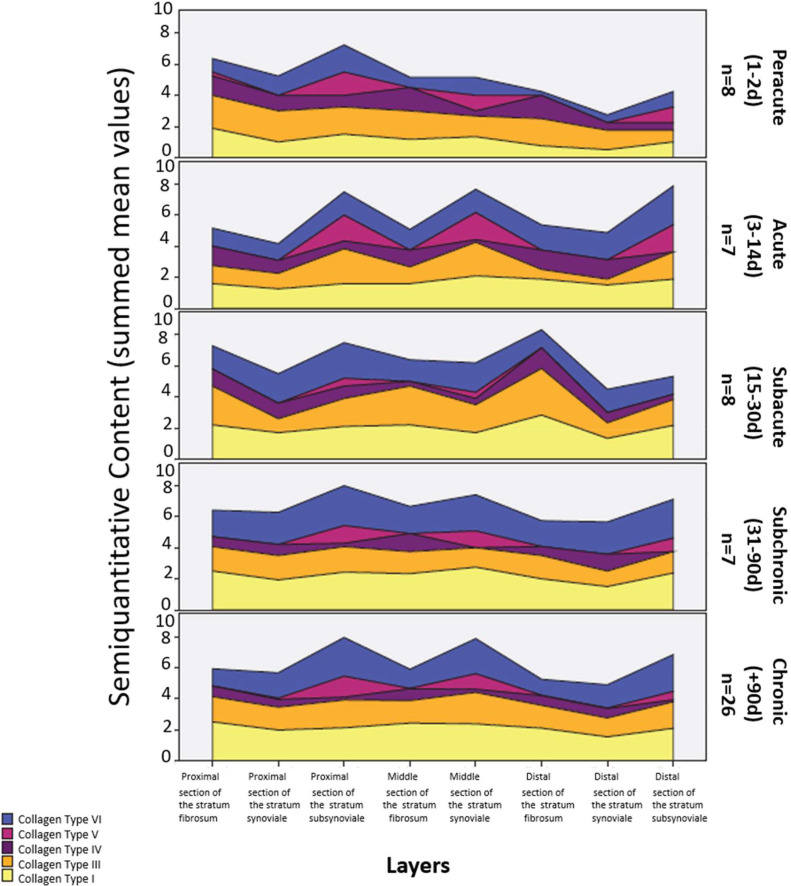
Semiquantitative content of the examined collagen types in relation to the lameness duration.

To evaluate the relationship between collagen expression and lameness duration, collagen type scores were averaged for each capsular layer, with the exception of the distal stratum fibrosum, which exhibited marked intra-layer variability. Significant associations were identified for collagen types I, III, and VI (Fig. 9). Overall, shorter lameness duration (peracute/acute) was associated with lower collagen type I and VI scores, whereas higher scores were observed in dogs with longer-lasting lameness (subchronic/chronic-progressive).

For collagen type I, dogs with peracute (1.59) and acute lameness (1.67) exhibited significantly lower scores than dogs with subchronic (2.25; *p* = 0.031 and *p* = 0.041, respectively; Bonferroni corrected) and chronic-progressive lameness (2.16; *p* = 0.030 and *p* = 0.033, respectively; Bonferroni corrected).

Collagen type III expression in the distal stratum fibrosum was significantly lower in dogs with acute lameness (0.63) compared with those with subacute lameness (3.00; *p* = 0.024, Bonferroni corrected).

Similarly, collagen type VI scores were significantly lower in dogs with peracute (1.12) and acute lameness (1.39) compared with those with subchronic lameness (2.07; *p* = 0.001, Bonferroni corrected).

Taken together, these findings demonstrate a progressive increase in collagen types I, III, and VI expression with longer duration of lameness. A comprehensive overview of significant differences in collagen type expression according to lameness duration is provided in Supplementary Table 6.

#### 3.3.4 Correlations of collagens regarding their localization

**Fig. 10:**
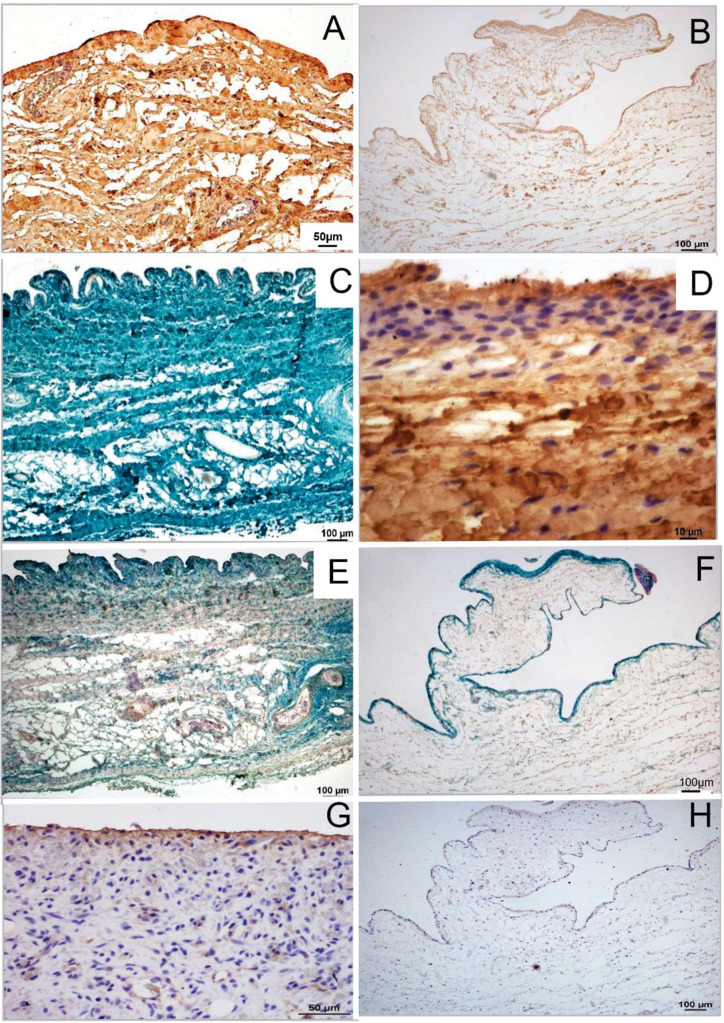
Visualization of different collagen types after immunohistochemistry in the stifle joint capsule. Original magnification: *A* × 20; B, C, E, F, and *H* × 10; *D* × 40; *G* × 30 A: Patellar luxation; dense fiber network of collagen type I (brown) throughout the stifle joint capsule (Ladewig staining). B: Femoral fracture; loosely arranged collagen type I (brown) in all three layers of the stifle joint capsule (Ladewig staining). C: Patellar luxation; collagen type III (blue-green) densely distributed across the entire stifle joint capsule (combined Weigert–Masson–Goldner staining). D: Cranial cruciate ligament rupture; collagen type III predominantly present in the synovial and fibrous layers (Ladewig staining). E: Patellar luxation; collagen type IV (brown) localized at the vascular basement membrane, with collagen type VI (blue-green) diffusely distributed in all three layers (Weigert–Volkmann–Strauss staining). F: Femoral fracture; collagen type IV (brown) sparsely present in blood vessels, with collagen type VI (blue-green) exclusively in the synovial layer (combined Weigert–Masson–Goldner staining). G: Patellar luxation; collagen type V primarily distributed in the synovial layer (hematoxylin–eosin staining). H: Femoral fracture; stifle joint capsule sample devoid of collagen type V (hematoxylin staining).

Collagen types exhibited distinct distribution patterns within the stifle joint capsule. Types I and III were prevalent throughout the cranio-lateral joint capsule, forming dense fiber bundles in all analyzed areas, making them the most frequently observed collagens (Fig. 10A-D). In contrast, collagen type IV was consistently localized to arterial and venous vessel walls, primarily within the stratum fibrosum, with lesser concentrations in the stratum subsynoviale and minimal presence in the stratum synoviale (Fig. 10E-F). Notably, collagen type V was uniquely found in the stratum synoviale in all animals except those with patellar luxation (Fig. 10G-H). Meanwhile, collagen type VI displayed a distribution pattern similar to that of types I and III but differed in quantity. A summary of the key distribution patterns and disease- and time-related changes for each examined collagen type is provided in Table 5.

**Table 5:** Summary of collagen type distribution patterns in the canine stifle joint capsule based on the results. Abbreviations: PL- Patellar luxation; CCLR-Cranial cruciate ligament rupture.

Collagen type	Predominant localization	Disease-associated changes	Association with lameness duration	Key interpretation
Type I	All capsule layers, especially stratum fibrosum and subsynoviale	Increased in PL, CCLR, and PL+CCLR compared to controls; altered I:III ratio in diseased joints	Significantly increased in subchronic and chronic–progressive lameness	Structural collagen reflecting capsular stiffening and chronic remodeling
Type III	All layers, often co-localized with type I	Increased relative to type I in PL+CCLR and CCLR	Increased with longer lameness duration (distal stratum fibrosum)	Marker of tissue remodeling and fibrosis
Type IV	Vessel walls (basement membranes)	Reduced or absent in synovial/subsynovial layers in PL+CCLR; higher in stratum fibrosum of controls	No clear association	Reflects vascular and basement membrane integrity
Type V	Predominantly stratum synoviale	Additional expression in proximal stratum subsynoviale exclusively in PL+CCLR	Increased in chronic conditions	Associated with capsular laxity and secondary fibrotic remodeling
Type VI	Pericellular matrix across layers	Highest in PL+CCLR; intermediate in PL and CCLR; lowest in controls	Significantly increased with longer lameness duration	Reflects chronic instability and extracellular matrix reorganization

#### 3.3.5 Correlation analysis of collagen types across different layers

For collagen type I, average score values across the three layers and sections showed strong correlations with one another (*p* < 0.05), with the exception of the middle section of the stratum subsynoviale, which did not correlate with any other section or layer.

A similar pattern was observed for collagen type III: the middle section of the stratum subsynoviale showed no correlation with scores from other layers (*p* < 0.05). Overall, collagen type III demonstrated moderate to strong correlations across most locations (*p* < 0.05), except for specific non-correlating pairs.

Notably, the proximal stratum fibrosum did not correlate with the middle stratum subsynoviale or the distal stratum synoviale (*p* < 0.05). Additionally, the middle stratum fibrosum did not correlate with the distal stratum synoviale or the distal stratum fibrosum (*p* < 0.05).

For collagen type IV, a significant correlation was found only between the middle stratum subsynoviale and the distal stratum fibrosum (*p* < 0.05). No other significant correlations were detected.

Collagen type V showed no correlation between the middle stratum subsynoviale and any other location. However, moderate correlations were observed with scores from the proximal stratum subsynoviale and both the proximal and distal sections of the stratum synoviale (*p* < 0.05). Moreover, collagen type V scores from the distal stratum subsynoviale correlated with all three sections of the stratum synoviale (*p* < 0.05).

Collagen type VI exhibited correlations across nearly all sections and layers, except for the proximal stratum synoviale and the distal stratum subsynoviale, where no significant correlations were observed (*p* < 0.05). Additionally, no section—other than the distal stratum fibrosum—showed correlation with scores from the stratum subsynoviale (*p* < 0.05).

## 4 Discussion

Patellar luxation (PL) and cranial cruciate ligament rupture (CCLR) represent significant orthopedic concerns in dogs, each characterized by a multifactorial etiology involving genetic predisposition, conformational abnormalities, and alterations in soft tissue structures (Wangdee et al., 2014; Farrell et al., 2015; Candela Andrade et al., 2020). Evidence suggests that connective tissue defects may contribute to increased joint laxity in PL cases (Paciello et al., 2003; Temwichitr et al., 2007). Considering the established association between collagen abnormalities and joint hyperextensibility in humans (Hildebrand et al., 2005), it is plausible that similar collagen-related defects may play a role in the pathogenesis of PL and CCLR, particularly within the relatively understudied stifle joint capsule.

However, it remains uncertain whether structural changes in the stifle joint capsule—including alterations in collagen composition or soft tissue laxity—represent primary defects contributing to PL, or instead reflect secondary adaptations resulting from skeletal malalignment and abnormal mechanical loading. Clarifying this relationship is crucial for a comprehensive understanding of the pathophysiology of both PL and CCLR.

Our previous research (Candela Andrade et al., 2025) demonstrated significant histomorphological changes associated with patellar luxation, such as variations in superficial cell layers, joint capsule villus formation, and absence of the stratum subsynoviale in chronic cases. Furthermore, we observed breed-dependent differences in joint capsule thickness. These findings underscore a critical gap in the current literature and strongly support the need for further research into potential collagen alterations within the different layers of the canine stifle joint capsule.

Our study reveals that collagen is the primary component of the extracellular matrix in the canine stifle joint capsule. Volkmann–Strauss staining shows that collagen fibers account for approximately 40% to 65% of the total area of the stifle joint capsule. The differences in collagen area fractions (%) are more pronounced among the individual layers of the joint capsule than between the proximal, middle, and distal sections. While the area fractions of these sections are significantly correlated, they differ notably across the three layers. Current literature does not categorize collagen content within joint capsule layers (Übeyli, 2006); instead, it typically reports dry mass content (mg/g). For example, tendon collagen—predominantly type I—is reported at approximately 810 to 850 mg/g (Krey et al., 1973). Übeyli (2006) estimates the collagen content of the joint capsule at about 900 mg/g. Additionally, Bey et al. (2005) note that the human shoulder joint capsule exhibits regionally distinct collagen fiber orientations.

In the stratum subsynoviale, we observed the largest area fractions of total collagen relative to a reference area of 0.33 mm², with collagen fibers occupying 61.11% in the proximal section and 64.41% in the distal section. This finding contrasts with Übeyli’s (2006) report on the human knee joint capsule, which states that the fibrous stratum contains the majority of the total collagen content. In our study, however, the stratum fibrosum exhibited only slightly smaller area fractions of total collagen compared to the subsynovial stratum. The stratum synoviale, known for its cell-rich composition (Geiler, 2013; Kung et al., 2015), had the lowest area fractions of total collagen, ranging from 42.93% in the middle section to 44.93% in the distal section. A significant correlation was also found between medium-sized and large dogs in the proximal section of the stratum fibrosum, where medium-sized dogs had 29.75% less total collagen. Due to a lack of comparable literature on collagen content within individual layers of joint capsules, regardless of species or joint type, no direct comparisons could be made.

We identified collagen types I, III, IV, V, and VI in the canine stifle joint capsule and found clear differences in their distribution within the capsule. The composition of collagen types and their relative abundance differed significantly among the three layers: stratum fibrosum, stratum subsynoviale, and stratum synoviale. Notably, the variation in collagen content was more pronounced between these layers than across the three anatomical sections (proximal, middle, distal). Collagen types I and III were ubiquitous throughout all three layers, appearing predominantly as densely packed fiber bundles. In dogs with patellar luxation, both collagen types were similarly abundant across all layers. However, in cases of patellar luxation accompanied by cruciate ligament rupture (PL+CCLR) and isolated cruciate ligament rupture (CCLR), we observed a significant increase in collagen type III relative to type I compared to the control group. This contrasting ratio of collagen type I to type III between acute and chronic stifle joint pathologies aligns with findings from previous studies (Brinckmann et al., 1999; Pingel et al., 2014). Similar patterns have been documented in human conditions such as lipodermatosclerosis and tendinopathies affecting the Achilles tendon (Pingel et al., 2014). Our findings regarding collagen types I and III are consistent with previous studies demonstrating fibrosis in the stifle joint capsule. Monument et al. (2012) showed that surgically induced contracture and immobilization in rabbits led to increased production of both collagen types I and III, mediated by mast cell–associated inflammation and subsequent collagen matrix remodeling. Steplewski et al. (2016) similarly reported increased collagen production and alterations in collagen texture in rabbit stifle joint capsules under comparable experimental conditions. Wong et al. (2015) further explored the gene regulatory response of the knee joint capsule in rats subjected to varying durations of immobilization. Their findings highlighted altered degradation of capsular matrix components, including various collagen types. These conditions resemble joint stiffness resulting from prolonged movement intolerance and secondary gonotrochosis observed in dogs with chronic stifle joint pathologies such as cruciate ligament rupture and patellar luxation.

Collagen type IV was confined to vessel walls, consistent with its known basement membrane localization (Plewig & Degitz, 2013). Collagen type V was predominantly localized in the stratum synoviale across all animal groups, except in dogs with patellar luxation and concomitant cranial cruciate ligament rupture (PL+CCLR). In this group, collagen type V was additionally detected in the proximal section of the stratum subsynoviale, significantly distinguishing it from all other groups.

This distribution pattern parallels the findings of Akhtar et al. (2012), who investigated collagen type V localization in the human shoulder joint capsule and reported its predominant presence within the stratum synoviale, as well as an association with capsular thinning and increased laxity. However, collagen type V might also be implicated in fibrotic remodeling processes of joint-associated soft tissues. In this context, the increased and expanded localization of collagen type V observed in dogs with PL+CCLR may reflect secondary remodeling of the synovial and subsynovial layers in response to chronic inflammation and altered mechanical loading, rather than representing a primary connective tissue defect.

Collagen type VI was most abundant in dogs with PL+CCLR, followed by those with either condition alone. The lowest levels were observed in dogs with femur fractures. Similar findings were reported by Boszczyk et al. (2001), who identified collagen types I, III, and VI in the joint capsule of human thoracic and lumbar intervertebral joints. In a subsequent study, Boszczyk et al. (2003) detected collagen types I, II, III, V, and VI in the posterior joint capsule of the human zygapophyseal joint, with increased levels of types I, III, and VI found in patients with progressive instability.

Regarding the duration of lameness, chronic conditions—specifically patellar luxation, cruciate ligament rupture, and their combination—showed a notable increase in collagen types V and VI. We observed significantly higher levels of collagen types I, III, V, and VI in cases of subchronic and chronic–progressive lameness compared to peracute and acute cases. Collagen types I and VI increased significantly throughout the whole joint capsule as lameness progressed, whereas collagen type III rose significantly only in the distal section of the stratum fibrosum. Similar studies have shown increased collagen expression in chronic joint diseases. A 1.5- to 2.5-fold increase in collagen types I, III, and V has been reported in human elbow joint capsules associated with chronic contracture (Hildebrand et al., 2005). Long-term collagen remodeling persisted for up to two years post-trauma in rabbit medial collateral ligaments (Hildebrand et al., 2004), and densification patterns were observed in human elbow capsules for up to 16 months post-injury (McFarland et al., 2002). Furthermore, Itoi & Hagiwara (2015) identified significant increases in collagen types I and III in cases of adhesive capsulitis (“frozen shoulder”). These results highlight the dynamic nature of collagen remodeling in chronic joint pathologies, suggesting that changes in collagen composition and organization occur over extended periods. Taken together, the association between increased collagen types I, III, V, and VI and longer lameness duration supports the interpretation that these changes predominantly reflect secondary extracellular matrix remodeling in response to prolonged inflammation and altered mechanical loading, rather than primary connective tissue defects.

From a translational perspective, the observed association between prolonged lameness duration and increased expression of collagen types I, III, V, and VI suggests that delayed surgical intervention may permit progressive extracellular matrix remodeling within the joint capsule. These findings support the concept that earlier correction of patellar luxation or cranial cruciate ligament instability could help limit secondary capsular fibrosis and stiffness, potentially improving postoperative joint mobility and outcome. In surgical procedures requiring capsular imbrication or partial capsular resection, histopathological evaluation of excised joint capsule tissue may provide additional information regarding disease chronicity and the extent of secondary remodeling. Such information could assist clinicians in postoperative prognosis and in anticipating the likelihood of persistent joint stiffness or reduced range of motion. Furthermore, the progressive increase in specific collagen types—particularly collagen types V and VI—in chronic conditions suggests that these molecules may have potential value as histological markers of disease chronicity or severity. While their use as clinical biomarkers would require further validation, the present findings indicate that collagen composition of the joint capsule reflects both the duration and mechanical burden of stifle joint pathology.

A major strength of the present study lies in the comprehensive, layer-specific characterization of collagen distribution within the canine stifle joint capsule, an aspect that has not previously been systematically evaluated. By combining quantitative morphometric assessment of total collagen area fractions with semiquantitative immunohistochemical evaluation of specific collagen types (I, III, IV, V, and VI), this study provides a multidimensional analysis of extracellular matrix organization. Furthermore, the inclusion of clinically well-characterized groups—patellar luxation, cranial cruciate ligament rupture, and combined pathology—together with stratification by lameness duration, enhances the translational relevance of the findings and allows assessment of remodeling patterns in relation to disease chronicity.

One important limitation of the present study concerns the method and anatomical location of sample collection. All joint capsule specimens were obtained exclusively from the lateral parapatellar region using a standardized parapatellar approach (Schebitz & Brass, 1999). This approach represents the only ethically and surgically justifiable access route in dogs undergoing treatment for cranial cruciate ligament rupture or medial patellar luxation, as additional or alternative capsular sampling would require unnecessary tissue trauma. While this ensured consistency across samples, it precluded evaluation of the medial joint capsule. This is particularly relevant given that all affected dogs exhibited medial patellar luxation, a condition associated with asymmetric mechanical loading of the joint capsule. In higher grades of patellar luxation, elongation and reduced tension of the lateral capsule may occur alongside increased stress and contracture on the medial side. Consequently, lateral-only sampling may underestimate or incompletely represent disease-associated capsular remodeling, especially in advanced cases where medial tension is greatest.

A further limitation relates to the small size of the control group, which reduces statistical robustness, increases susceptibility to outliers, and may limit the generalizability of comparisons with diseased samples. Control joint capsule samples were obtained from dogs with distal femoral fractures or from animals euthanized for severe, non-orthopedic systemic disease. Orthopedic disease affecting the stifle joint was excluded based on clinical history, orthopedic examination, and owner-reported information; however, fracture-associated inflammatory responses may influence local connective tissue remodeling. In addition, while one control dog suffered from end-stage chronic kidney disease, there is currently no specific evidence in the veterinary literature demonstrating a direct effect of renal disease on joint capsule collagen composition. Nevertheless, a potential indirect influence of systemic illness on connective tissue metabolism cannot be entirely excluded and should be considered when interpreting differences between control and diseased groups, particularly for collagen types IV and V. The collection of genuinely healthy stifle joint capsule samples poses considerable ethical and practical challenges, as owners are often unwilling to consent to tissue collection following euthanasia. For a more reliable baseline, future investigations should ideally incorporate specimens from clinically healthy dogs, preferably matched for age. However, such sampling is difficult to achieve due to these ethical and logistical limitations. Collaborative efforts with other institutions may therefore represent a valuable strategy to improve access to appropriately matched control samples.

Age matching between control and diseased groups represents an additional limitation. The age range of the control animals was wide, spanning from juvenile to geriatric dogs, which limits direct comparability with the affected population. Age-related alterations in collagen turnover and organization may therefore have contributed to variability within the control group and potentially influenced baseline collagen distribution patterns. Obtaining truly healthy stifle joint capsule tissue from clinically normal dogs is both ethically and practically challenging, and owners are often reluctant to consent to post-mortem tissue harvesting. These constraints significantly limit access to ideal control material in veterinary orthopedic research. In addition, demographic variables such as breed, body size, and sex distribution were not strictly matched between groups, which may represent additional sources of biological variability.

Another limitation of this study is that, although semiquantitative immunohistochemical scoring was performed independently by two observers with consensus review in discrepant cases, formal inter-observer reliability statistics were not calculated. While predefined scoring criteria and consensus evaluation were applied to enhance consistency, the absence of quantitative agreement metrics may limit the reproducibility and external comparability of the semiquantitative assessment across independent observers or future studies.

An additional conceptual limitation relates to the cross-sectional design of the study. Although the observed alterations in collagen types I, III, V, and VI were associated with clinical duration and joint instability, the data do not allow definitive conclusions regarding causality or a clear distinction between primary structural abnormalities and secondary adaptive remodeling processes. The identified collagen patterns are consistent with progressive extracellular matrix remodeling in response to chronic inflammation and altered biomechanical loading; however, the present dataset does not permit confirmation of inherent connective tissue defects or congenital capsular abnormalities as initiating factors. Therefore, interpretations suggesting a primary structural predisposition should be made with caution.

Despite these constraints, the consistent and disease-specific collagen distribution patterns observed across patellar luxation and cranial cruciate ligament rupture groups, in contrast to controls, indicate that the alterations are likely associated with chronic joint pathology rather than solely attributable to confounding factors. Future studies incorporating age-matched control animals, bilateral sampling of medial and lateral capsule regions, and stratification by patellar luxation grade are warranted to further clarify the relationship between mechanical loading, disease chronicity, and collagen remodeling in the canine stifle joint capsule

## 5 Conclusions

Our study identifies significant histological differences in the stifle joint capsule of dogs with congenital patellar luxation compared to healthy controls, particularly in terms of collagen composition. These findings indicate that alterations in collagen composition of the stifle joint capsule are associated with patellar luxation and cranial cruciate ligament rupture, and likely reflect a combination of predisposing structural factors and secondary remodeling processes driven by chronic inflammation and mechanical overload. Variations in collagen types (I, III, IV, V, and VI) and their abundances across different joint capsule layers provide novel insights into the pathogenesis of patellar luxation, highlighting a dynamic remodeling response to injury. The histological insights gained from this research enhance our understanding of joint disorders such as patellar luxation and ligament injuries. This knowledge is essential for developing targeted treatment strategies that can improve clinical outcomes and prevent further joint damage.

Overall, this study emphasizes the importance of ongoing research into the histomorphological aspects of canine joint disorders. Such investigations are essential for advancing treatment methodologies and refining breeding practices, ultimately contributing to improved health outcomes and quality of life for affected dogs.

## Funding

This research did not receive any specific grant from funding agencies in the public, commercial, or not-for-profit sectors.

## Data availability

The original data supporting the findings of this study are included in the article and its Supplementary Materials. Further inquiries can be directed to the corresponding author.

## Ethical statement

Ethical approval for the study “Histological analysis of collagen composition in the stifle joint capsule of dogs with congenital patellar luxation and cranial cruciate ligament rupture” was not required in accordance with the German Animal Welfare Act. The Landesamt für Gesundheit und Soziales Berlin reviewed the study and confirmed that it does not qualify as an animal experiment under § 7 (2) (Study Registration Number: StN 032/25). All tissue samples were obtained exclusively from client-owned dogs undergoing medically indicated and established surgical treatments for patellar luxation or cranial cruciate ligament rupture. Joint capsule samples were collected during routine capsular resection and capsular tightening procedures, which are integral components of standard surgical management for these conditions. No additional interventions or procedures causing pain, suffering, or harm were performed for research purposes. Control samples were obtained from dogs euthanized for unrelated medical reasons. Written informed consent was obtained from all owners for the use of their animals’ tissue samples in this study.

## CRediT authorship contribution statement

**Mario Candela Andrade:** Writing – review & editing, Methodology, Funding acquisition, Formal analysis, Data curation. **Petra Peer:** Writing – review & editing, Writing – original draft, Methodology, Investigation, Formal analysis, Data curation, Conceptualization. **Pavel Slunsky:** Writing – review & editing, Supervision. **Matias Aguilera-Rojas:** Writing – review & editing, Data curation. **Johanna Plendl:** Writing – review & editing, Supervision. **Leo Brunnberg:** Writing – review & editing, Supervision.

## Declaration of competing interest

The authors declare that they have no known competing financial interests or personal relationships that could have appeared to influence the work reported in this paper.

## Appendix Supplementary materials

Image, application 1
