# Supplementary material for: Histological analysis of collagen composition in the stifle joint capsule of dogs with congenital patellar luxation and cranial cruciate ligament rupture
Source: Vet Anim Sci. 2026 Mar 26;32:100635. doi: 10.1016/j.vas.2026.100635 (PMC13084655; doi:10.1016/j.vas.2026.100635)
Supplement: Supplementary file 1 [file mmc1.docx]

**Table 1**: Frequencies of canine stifle joint capsule samples and conditions among various breeds groups

| **Breed Group** | **Breed** | **Patellar Luxation (PL)** | **Cruciate Ligament Rupture (CCLR)** | **PL + CCLR** | **Control group** | **Sample Count (n)** |
| --- | --- | --- | --- | --- | --- | --- |
| Toy breeds  (0-5kg) | Chihuahua | 10 | 0 | 0 | 1 | 11 |
|  | Chihuahua Mix | 0 | 1 | 0 | 0 | 1 |
|  | Russkiy Toy | 1 | 0 | 0 | 0 | 1 |
|  | Prager Rattler | 2 | 0 | 0 | 0 | 2 |
| Small breeds  (5-10kg) | Rehpinscher | 1 | 0 | 0 | 0 | 1 |
|  | Rehpinscher Mix | 1 | 0 | 0 | 0 | 1 |
|  | Yorkshire Terrier | 5 | 0 | 1 | 0 | 6 |
|  | Yorkshire Terrier Mix | 1 | 0 | 0 | 0 | 1 |
|  | Maltese | 2 | 0 | 1 | 0 | 3 |
|  | Bolonka Zwetna | 2 | 0 | 0 | 0 | 2 |
|  | Shih Tzu | 0 | 1 | 0 | 0 | 1 |
|  | Pekingese | 1 | 0 | 0 | 0 | 1 |
|  | Chinese Crested Dog | 1 | 0 | 0 | 0 | 1 |
|  | Cavalier King Charles Spaniel | 2 | 0 | 0 | 0 | 2 |
|  | Miniature Poodle | 2 | 0 | 0 | 1 | 3 |
|  | Boston Terrier | 1 | 0 | 0 | 0 | 1 |
|  | West Highland White Terrier | 3 | 0 | 1 | 0 | 4 |
|  | Mixed Breed < 10 kg | 6 | 0 | 0 | 1 | 7 |
| Medium-Sized breed  (10-20kg) | Shetland Sheepdog | 1 | 0 | 0 | 0 | 1 |
|  | Shetland Sheepdog Mix | 1 | 0 | 0 | 0 | 1 |
|  | French Bulldog | 1 | 0 | 0 | 0 | 1 |
|  | Tibetan Terrier | 1 | 0 | 0 | 0 | 1 |
|  | Tibetan Terrier Mix | 0 | 1 | 0 | 0 | 1 |
|  | Beagle | 0 | 1 | 0 | 0 | 1 |
|  | Border Collie | 1 | 0 | 0 | 0 | 1 |
|  | Collie Mix | 0 | 1 | 0 | 0 | 1 |
|  | Basset Fauve de Bretagne | 1 | 0 | 0 | 0 | 1 |
|  | Shar Pei | 2 | 0 | 0 | 0 | 2 |
|  | Podenco Canario Mix | 1 | 0 | 0 | 0 | 1 |
|  | Mixed Breed 10 - 20 kg | 5 | 1 | 1 | 0 | 7 |
| Large breeds  >20kg | German Shepherd Mix | 1 | 0 | 0 | 0 | 1 |
|  | Boxer | 0 | 2 | 0 | 0 | 2 |
|  | Akita Inu | 1 | 0 | 0 | 0 | 1 |
|  | American Staffordshire Terrier | 1 | 0 | 0 | 1 | 2 |
|  | Caucasian Ovcharka Mix | 0 | 1 | 0 | 0 | 1 |
|  | Leonberger | 0 | 1 | 0 | 0 | 1 |
|  | Great Dane | 0 | 1 | 0 | 0 | 1 |
|  | Mixed Breed > 20 kg | 1 | 0 | 0 | 0 | 1 |

**Table 2**- Step-by-step process for the preparation of slides for histological staining, ensuring consistent and high-quality tissue samples for analysis. Paraplast®, Fa. Shandon Labortechnik GmbH, Frankfurt, Germany. Jung Histoslide 2000R®, Leica Instruments GmbH, Nussloch, Germany. HistoBond®, Marienfeld, Lauda-Königshofen, Germany. Eurocassette®, Fa. Reichert-Jung, Heidelberg, Germany.

| **Step** | **Process** | **Details** |
| --- | --- | --- |
| 1. Sample Collection | Surgical or post-mortem | Laterally parapatellar, 0.5-2 cm² size with scalpel (Aesculap®, USA). Attention was paid to the vastus lateralis muscle, which extends proximally into the joint capsule. The primary considerations for this biopsy location were to ensure good conditions for the healing process and to tighten the joint capsule (Schebitz H and Brass 1999) |
| 2. Fixation | 4% formaldehyde solution | 55 to 60 hours at +4°C. The fixative volume was 20 times that of the sample to ensure complete penetration. Accordingly, large containers were used to allow the joint capsule biopsies to float freely. For this study, tissue samples were fixed for approximately 55 to 60 hours to ensure reliability. |
| 3. Washing | Running tap water | 3 hours to remove fixative in standard plastic cassettes (Eurocassette®). The absence of formaldehyde was confirmed by adding Schiff's reagent. |
| 4. Dehydration | Ascending alcohol series | 50% to 100% alcohol, 2-4 hours each step |
| 5. Clearing | Xylene | 2-4 hours total, in two portions |
| 6. Embedding | Paraffin (Paraplast®) | 60°C, 2 hours total. Finally, the joint capsule biopsies were individually hand-cast into 2 x 3 cm metal molds. |
| 7. Sectioning | Sled microtome (Jung Histoslide 2000®) | 4 μm thick sections |
| 8. Mounting | Silanized slides (Histobond®) | Water bath stretching at 40°C |
| 9. Adhesion | Custom adhesive | 1:1 commercial glue and Poly-L-Lysine |
| 10. Drying | Incubator | 37-45°C for at least 12 hours |
| 11. Deparaffinization | Xylene and descending alcohol series | Xylene (2x10 min), 100% Ethanol (2x3 min), 96% to 70% Ethanol (1x3 min each). Rehydration in distilled water for 1-2 minutes. |

**Table 3:** Concentrations of the antibodies with the corresponding recommended dilutions and the optimized dilutions used for the canine joint capsule samples.

| **Primary antibody** | **Antiserum concentration** | **Recommended dilution** | **Dilution used in this study** | **Secondary antibody (concentration, dilution)** |
| --- | --- | --- | --- | --- |
| Polyclonal rabbit anti-collagen I (Abcam, ab34710) | 1.00 mg/mL | Not specified | 1:50 | Donkey anti-rabbit IgG (Abcam), 0.5 mg/mL, 1:300 |
| Polyclonal rabbit anti-collagen III (Abcam, ab7778) | 1.00 mg/mL | 1:1000 | 1:100 | Donkey anti-rabbit IgG (Abcam), 0.5 mg/mL, 1:300 |
| Polyclonal rabbit anti-collagen IV (Abcam, ab6586) | 1.00 mg/mL | 1:500 | 1:20 | EnVision+ System-HRP labelled polymer anti-mouse (Dako), ready-to-use |
| Polyclonal rabbit anti-collagen V (Abcam, ab7046) | 1.02 mg/mL | 1:500 | 1:50 | Donkey anti-rabbit IgG (Abcam), 0.5 mg/mL, 1:300 |
| Polyclonal rabbit anti-collagen VI (Abcam, ab6588) | 1.00 mg/mL | Not specified | 1:100 | Donkey anti-rabbit IgG (Abcam), 0.5 mg/mL, 1:300 |

**Table 4**. Breed-associated differences in collagen type distribution across capsular layers

Significant differences in collagen type distribution among toy, small, medium-sized, and large dogs are summarized. Values represent semiquantitative immunohistochemical scores for collagen types I–VI within the specified capsular regions. Only statistically significant pairwise comparisons are shown. Statistical analysis was performed using non-parametric Mann–Whitney U tests for independent samples, with confirmation by one-way ANOVA. P-values were adjusted using Bonferroni correction, and significance was set at p < 0.05. Abbreviations: SF, stratum fibrosum; SS, stratum subsynoviale; SYN, stratum synoviale; Prox, proximal; Mid, middle; Dist, distal.

| **Region** | **Collagen Type** | **Comparison** | **Values** | **p-value (Bonferroni)** |
| --- | --- | --- | --- | --- |
| Proximal SF | Type I | Toy > Small | 2.72 vs 1.88 | 0.050 |
| Proximal SS | Type I | Toy > Large | 2.39 vs 0.58 | 0.011 |
| Proximal SS | Type I | Medium > Large | 2.00 vs 0.58 | 0.011 |
| Distal Synoviale | Type III | Medium > Large | 1.71 vs 0.36 | 0.016 |
| Distal Synoviale | Type III | Toy > Large | 2.19 vs 0.38 | 0.047 |
| Proximal SF | Type I | Small < Toy & Medium | 1.88 vs 2.72 / 2.75 | 0.007 |
| Middle SF | Type I | Small < Medium | 1.87 vs 2.66 | 0.028 |

**Table 5:** Significant differences in collagen type V expression in the proximal stratum subsynoviale across different disease states

| **Region** | **Comparison** | **Collagen V Score** | **p-value (Bonferroni corrected)** |
| --- | --- | --- | --- |
| Proximal stratum subsynoviale | PL+CCLR vs. PL | 0.33 vs. 0.01 | 0.001 |
| Proximal stratum subsynoviale | PL+CCLR vs. Controls | 0.33 vs. 0.00 | 0.002 |

**Table 6:** Significant differences in collagen type expression according to lameness duration

| **Collagen Type** | **Capsular Region** | **Comparison (Lameness Duration)** | **Scores** | **p-value (Bonferroni corrected)** |
| --- | --- | --- | --- | --- |
| Type I | Mean across capsular layers | Peracute vs. Subchronic | 1.59 vs. 2.25 | 0.031 |
| Type I | Mean across capsular layers | Peracute vs. Chronic-progressive | 1.59 vs. 2.16 | 0.030 |
| Type I | Mean across capsular layers | Acute vs. Subchronic | 1.67 vs. 2.25 | 0.041 |
| Type I | Mean across capsular layers | Acute vs. Chronic-progressive | 1.67 vs. 2.16 | 0.033 |
| Type III | Distal stratum fibrosum | Acute vs. Subacute | 0.63 vs. 3.00 | 0.024 |
| Type VI | Mean across capsular layers | Peracute vs. Subchronic | 1.12 vs. 2.07 | 0.001 |
| Type VI | Mean across capsular layers | Acute vs. Subchronic | 1.39 vs. 2.07 | 0.001 |
